# Proteomics Analysis with a Nano Random Forest Approach Reveals Novel Functional Interactions Regulated by SMC Complexes on Mitotic Chromosomes*[Fn FN2]

**DOI:** 10.1074/mcp.M116.057885

**Published:** 2016-05-26

**Authors:** Shinya Ohta, Luis F. Montaño-Gutierrez, Flavia de Lima Alves, Hiromi Ogawa, Iyo Toramoto, Nobuko Sato, Ciaran G. Morrison, Shunichi Takeda, Damien F. Hudson, Juri Rappsilber, William C. Earnshaw

**Affiliations:** From the ‡Center for Innovative and Translational Medicine, Medical School, Kochi University Kohasu, Oko-cho, Nankoku, Kochi 783–8505, Japan;; §Wellcome Trust Centre for Cell Biology, School of Biological Sciences, University of Edinburgh, Mayfield Road, Edinburgh EH9 3BF, UK;; ¶Centre for Chromosome Biology, School of Natural Sciences, National University of Ireland Galway, Galway, Ireland;; ‖Department of Radiation Genetics, Kyoto University Graduate School of Medicine, Yoshida Konoe, Sakyo-ku, Kyoto 606–8501, Japan;; **Murdoch Childrens Research Institute, Royal Children's Hospital, Melbourne, Victoria 3052, Australia;; ‡‡Chair of Bioanalytics, Institute of Biotechnology, Technische Universität Berlin, 13355 Berlin, Germany

## Abstract

Packaging of DNA into condensed chromosomes during mitosis is essential for the faithful segregation of the genome into daughter nuclei. Although the structure and composition of mitotic chromosomes have been studied for over 30 years, these aspects are yet to be fully elucidated. Here, we used stable isotope labeling with amino acids in cell culture to compare the proteomes of mitotic chromosomes isolated from cell lines harboring conditional knockouts of members of the condensin (SMC2, CAP-H, CAP-D3), cohesin (Scc1/Rad21), and SMC5/6 (SMC5) complexes. Our analysis revealed that these complexes associate with chromosomes independently of each other, with the SMC5/6 complex showing no significant dependence on any other chromosomal proteins during mitosis. To identify subtle relationships between chromosomal proteins, we employed a nano Random Forest (nanoRF) approach to detect protein complexes and the relationships between them. Our nanoRF results suggested that as few as 113 of 5058 detected chromosomal proteins are functionally linked to chromosome structure and segregation. Furthermore, nanoRF data revealed 23 proteins that were not previously suspected to have functional interactions with complexes playing important roles in mitosis. Subsequent small-interfering-RNA-based validation and localization tracking by green fluorescent protein-tagging highlighted novel candidates that might play significant roles in mitotic progression.

Mitotic chromosome condensation, regulated sister chromatid cohesion, and chromosome interactions with the spindle are crucial to ensuring appropriate genome segregation during mitosis and meiosis. The first 2 of these events require the activity of protein complexes containing structural maintenance of chromosomes (SMC)^1^ proteins. SMC proteins are large polypeptides that fold back upon themselves via a central hinge region, enabling the formation of a long, antiparallel coiled-coil domain (1). ATP binding to a bipartite adenosine triphosphate (ATP)-binding cassette ATPase motif juxtaposes the N- and C-terminal globular domains of each SMC protein, forming a closed loop. A strap-like kleisin protein then holds the heads of both SMC proteins together. Although the exact role of the ATPase activity is unknown, it is essential for condensin function (2–4).

The SMC complexes can be divided into 3 groups. The first and most-studied SMC complex is cohesin, which contains SMC1, SMC3, the kleisin Scc1, and 1 of 3 auxiliary different subunits (SA1-SA3; Scc3 in budding yeast) (5–7). Cohesin links sister chromatids together until the kleisin subunit is subsequently cleaved by a protease, which triggers the onset of anaphase chromosome movements. The predominant view is that cohesin holds sister chromatids together by encircling daughter DNA molecules during DNA replication (8, 9). In addition to its role in mitosis, cohesin has also been reported to play roles in interphase chromosome organization, transcription, and DNA repair (1, 10).

The second SMC complex, condensin, is a pentamer containing a dimer of SMC2 and SMC4. In condensin I, the SMC subunits associate with the kleisin subunit CAP-H plus the auxiliary subunits CAP-G and CAP-D2. An alternate complex, condensin II, contains the same SMC2/SMC4 dimer complexed with CAP-H2, CAP-G2, and CAP-D3 (2–4, 11–14). Although the requirement for condensin function in chromosome architecture has been well established, its mechanism of action remains an open question.

Condensin can supercoil DNA in an ATP-dependent reaction (3–7) and can promote DNA annealing without the need for ATP (8, 9, 15, 16). Recent evidence suggests that yeast condensin, like cohesin, may function by encircling chromatin fibers (17), although in isolated condensin, the coiled coils have a closed rod-like structure (1). The roles of all of these processes in mitotic chromosome formation remain unclear. Condensin also regulates the association of other nonhistone chromatin proteins with mitotic chromosomes by an unknown mechanism (18, 19). Although near-normal chromatin compaction can be achieved in vertebrate chromosomes ≥95% depleted of condensin, this organized chromosomal architecture is lost during anaphase when protein phosphatase 1 is targeted by Repo-Man to the separating chromatids (20). An emerging view is that the chromokinesin KIF4A collaborates with condensin I to promote the lateral compaction of chromatid arms, whereas condensin II and DNA topoisomerase IIα promote the shortening of chromatid axes (21–23).

The function of the third SMC complex is less clear, and it is known simply as the SMC5/6 complex. In budding yeast, this complex consists of SMC5, SMC6, and the non-SMC elements NSE1–6 (24, 25). NSE2 is an E3 ligase for small ubiquitin-like modifiers. The vertebrate SMC5/6 complex was recently suggested to contain homologs of NSE1–4. However, the organization of the complex remains less clear (26). The SMC5/6 complex has been implicated in DNA repair and recombination (27–31) and for the resolution of chromatin links during meiosis (25, 32, 33).

A limiting factor that hinders functional insights obtained from recent large-scale proteomics studies is the difficulty in recognizing functional relationships hidden in large data sets. Multivariate profiling employing principal component analysis has proven useful (34), as has the multiclassifier combinatorial proteomics approach for integrating data from multiple “classifiers” using Random-Forest analysis (35). This latter approach appeared to be particularly useful in distinguishing cytoplasmic “hitchhikers” associated with isolated mitotic chromosomes from proteins that contribute to chromosomal structure and segregation during mitosis (35, 36).

Here, we analyzed the role of the 3 SMC protein complexes in mitotic chromosome protein compositions using a novel nano Random Forest (nanoRF) analysis. NanoRF analysis was developed from multiclassifier combinational proteomics with the aim of using targeted machine learning to integrate proteomic data from the analysis of different mutants that affect specific protein complexes (37). This analysis revealed that SMC complexes act independently of each other, with only weak dependence between condensin I and condensin II. Condensin depletions appeared to have the most profound effects on the mitotic chromosome proteome, and depletions of the SMC5/6 complex appeared to have the least impact, with the only significant changes occurring exclusively among members of that complex. This analysis revealed few structural links between cohesin and condensin, in contrast to suggestions of several published studies (14, 38). Finally, our nanoRF analysis implied functional links between condensin II and the kinetochore that were not readily apparent by simple inspection of the proteomics data. This information was subsequently used to predict and confirm proteins that affect mitotic progression.

## EXPERIMENTAL PROCEDURES

### 

#### 

##### Cell Culture

Wild-type DT40 cells (clone 18), as well as SMC2, CAP-H, CAP-D3, Scc1, or SMC5 conditional knockouts, were maintained in Roswell Park Memorial Institute (RPMI) 1640 medium (Wako Pure Chemical Industries Ltd., OSAKA) supplemented with 10% (v/v) fetal bovine serum (FBS), 1% calf serum, 100 U/ml penicillin, and 100 μg/ml streptomycin (Wako Pure Chemical Industries Ltd.) at 39 °C in a humidified incubator with an atmosphere containing 5% CO_2_ (19, 39–41). For ^13^C and ^15^N labeling of Lys and Arg, cells were maintained in RPMI without L-lysine and L-arginine (Thermo Fisher Scientific, Waltham, MA) supplemented with 10% (v/v) FBS dialyzed against a 10,000-molecular-weight cut-off membrane (Sigma-Aldrich, St. Louis, MO), 100 μg/ml ^13^C_6_, ^15^N_2_-L-lysine:2HCl, 30 μg/ml ^13^C_6_, ^15^N_4_-l-arginine:HCl (Wako Pure Chemical Industries Ltd.), 100 U/ml penicillin, and 100 μg/ml streptomycin (Gibco-BRL; Thermo Fisher Scientific) at 37 °C in a humidified incubator with an atmosphere containing 5% CO_2_. To generate SMC2^OFF^, CAP-H^OFF^, CAP-D3^OFF^, Scc1^OFF^, or SMC5^OFF^ cells, we grew SMC2^ON/OFF^ (19), CAP-H^ON/OFF^, CAP-D3^ON/OFF^ (39), Scc1^ON/OFF^ (41), or SMC5^ON/OFF^ (40) cells in the presence of doxycycline for 30, 26, 24, 19, or 60 h, respectively, prior to blocking with nocodazole to inhibit expression. HeLa and U2OS cells in the exponential growth phase were seeded onto coverslips and grown overnight in Dulbecco's Modified Eagle's Medium (DMEM) supplemented with 10% FBS at 37 °C in an atmosphere containing 5% CO_2_.

##### Mitotic Chromosome Isolation

DT40 cells were incubated with nocodazole for 13 h, resulting in a mitotic index of 70–90%. Mitotic chromosomes were isolated using a buffer system containing polyamines and ethylenediaminetetraacetic acid optimized for mitotic chromosome isolation from DT40 cells (35, 42). Five OD_260_ units were obtained by pooling 1.0 × 10^9^ DT40 cells from four independent preparations and solubilizing them in sodium dodecyl sulfate-polyacrylamide gel electrophoresis (SDS-PAGE) sample buffer.

##### Experimental Design and Statistical Rationale

Each sample for mass spectrometry (MS) analysis was generated by combining three individual preparations of isolated mitotic chromosomes. Three biological replicates for Scc1 KO cells, or two biological replicates for SMC2, CAP-H, CAP-D3, or SMC5 KO cells were analyzed (supplemental Fig. S3).

##### MS Analysis

Proteins were separated into high- and low-molecular weight fractions by SDS-PAGE, in-gel digested using trypsin (43), and separated into 30 fractions each using strong cation-exchange chromatography (SCX). The individual SCX fractions were desalted using StageTips (44) and analyzed by liquid chromatography-MS on an LTQ-Orbitrap (Thermo Fisher Scientific) coupled to high-performance liquid chromatography via a nanoelectrospray ion source. The six most intense ions of each full mass spectrum acquired in the Orbitrap analyzer were fragmented and analyzed in the linear-ion trap. The MS data were analyzed using MaxQuant 1.4.0.3 for generating peak lists, searching peptides, and protein identification (45), using the UniProt *Gallus gallus* protein database (release 2013_07 containing 17598 entries) and our in-house chicken database as a reference (supplemental Table S1). The precursor mass tolerance was set to 20 parts per million (ppm) for the first search and 6 ppm for the main search. Fragment ions were searched with a mass tolerance of 20 ppm. A minimum of 2 razors (unique) peptides and a false-discovery rate (FDR) of 1% were required for protein quantification. The MS proteomics data and all parameters used in MaxQuant were deposited in the ProteomeXchange Consortium (http://proteomecentral.proteomexchange.org) via the PRIDE partner repository under the data set identifier PXD003427 (46, 47). The annotated spectra are able to be accessed in MS-viewer (http://prospector2.ucsf.edu/prospector/cgi-bin/msform.cgi?form=msviewer) with the search key rekeqln3ua (48).

##### Calculation of dependences

To calculate the dependences of each mitotic chromosomal-associating protein, we averaged the SILAC ratio of the individual experiments using the following formula:

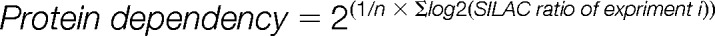


##### NanoRF Analysis

NanoRF analysis is based on the supervised algorithm Random Forests (http://stat-www.berkeley.edu/users/breiman/RandomForests) and the R RandomForest package (http://cran.r-project.org/web/packages/randomForest/). The nanoRF tool package is available from the Github repository (https://github.com/EarnshawLab/nanoRF). The missing values were replaced by the median SILAC value of the experiment. We assessed the classification/fractionation quality by receiver operating characteristic (ROC)-curve analysis and the Matthews correlation coefficient (MCC).

##### Mitotic Indices of Small Interfering RNA (siRNA)-transfected Human Cells

All siRNA oligos used in this study were purchased as siGENOME SMARTpools to avoid off-target effects (Thermo Fisher Scientific; PPP2R5C, M009433–01; PHF2, M012912–01; PPP2R5A, M009352–01; KMT2A, M009914–01; SMARCA1, M011392–01; PPP2R5D, M009799–01; VRK1, M004683–02; CISD2, M032593–00; CYP51A1, M009215–01; ACO1, M010037–00; LNPEP, M005926–02; GTSE1, M005286–01; PPP2R2A, M004824–01; ZNF512B, M013934–00; PTPN6, M009778–02; NNT, M009809–01; SPDL1, M016970–01; AICDA, M021409–01; INTS8, M020270–01; SNX9, M017335–02; CCNB3, M003208–03; CXorf57, M014634–01; HMGXB4, M018122–01; BACH2, M009787–00; ZFC3H1, M020839–01; KIF20A, M004957–01; and C15orf24, M022219–01). Each siRNA pool (40 nm concentration in the supernatant) was administered to HeLa cells (Kyoto Pharmaceutical Industries, Ltd., Kyoto, Japan) maintained in DMEM supplemented with 10% (v/v) FBS, 100 U/ml penicillin, and 100 μg/ml streptomycin (Wako Pure Chemical Industries, Ltd.) at 37 °C in a humidified incubator with an atmosphere containing 5% CO_2_. Cells were transfected with siRNAs at 60% confluence using Lipofectamine RNAi MAX (Life Technologies; Thermo Fisher Scientific) in complete medium, without antibiotics. Cells were maintained in this medium for 72 h, and nocodazole or taxol was added 14 h before the cells were harvested. Cells were washed once in phosphate-buffered saline (PBS) with 1% bovine serum albumin (BSA) and fixed in 70% ethanol. The fixed cells were washed again in PBS and probed with antihistone H3 phospho-S10 (1:1000, 06–570; Millipore, Darmstadt, Germany) and goat antirabbit IgG (Alexa Fluor 488; 1:1000, A-21210, Life Technologies; Thermo Fisher Scientific) antibodies. Cells were treated with propidium iodide (PI) and RNase A for 30 min and analyzed by flow cytometry using a FACSCalibur instrument (BD Biosciences, Franklin Lakes, NJ).

##### Transfection of Plasmid DNA and Indirect Immunofluorescence Microscopy

Halo-tagged VRK1 was provided by the Kazusa DNA Research Institute (FHC11229; Promega, Madison, WI). PTPN6 cDNA (IRAL004E22; http://dna.brc.riken.jp/) was provided by RIKEN BRC through the National Bio-Resource Project of Japan (49). Expression vectors were transfected into U2OS cells at 60–80% confluence using Lipofectamine LTX (Invitrogen, Carlsbad, CA) in complete medium, without antibiotics. Cells were maintained in this medium for 24 h. Cells transfected with expression vectors encoding Halo-tagged VRK1 were incubated with 5 μm HaloTag Oregon Green Ligand (G2801; Promega) for 2 h and washed in DMEM without the Halo-tag ligand. Cells were fixed for 5 min with 4% (v/v) paraformaldehyde (Wako Pure Chemical Industries Ltd.) in PBS. After permeabilization with PBS containing 0.15% (v/v) Triton X-100, coverslips were blocked with PBS containing 1% (v/v) BSA. Cells were probed with antibodies against α-tubulin (B512, 1:2000, mouse; Sigma-Aldrich) for 30 min. Cells were washed 3 times in PBS for 5 min and incubated with Alexa594-labeled secondary antibodies at a 1: 600 dilution for 30 min. DNA was counterstained using 4′, 6-diamidino-2-phenylindole at 0.1 μg/ml. Single-confocal plane images were obtained using an Olympus FV1000 microscope, which is based on an IX81 confocal microscope system, with a UPlanSApo 60×/1.35 oil-immersion objective lens (Olympus) and FV10-SAW2.1 software (Olympus). The images were Kalman-filtered to suppress background noise.

## RESULTS

### 

#### 

##### Dependence of the Mitotic Chromosome Proteome on Condensins I and II

Previous findings revealed that although SMC2-depleted chromosomes appeared relatively normal during early mitosis, they lost their organized structure during anaphase (20). Recent proteomics data from our group showed an apparent loss of almost 50% of the core histones in SMC2-depleted chromosomes isolated from synchronized nocodazole-arrested cells at early mitosis (35). Investigation of this finding revealed a flaw when standard SILAC protocols were used for these samples.

The decreased levels of core histones (histones H2A, H2B, H3, and H4) in mitotic chromosomes isolated from SMC2-knockout cells could be explained by 2 possibilities. First, the binding of core histones to mitotic chromosomes might be reduced in SMC2^OFF^ cells. However, micrococcal nuclease digestion of chromosomes isolated from cells both with and without SMC2 expression revealed similar nucleosomal “ladders” with repeat lengths of ∼200 bp (supplemental Fig. S1). This finding was suggestive of a similar nucleosome density in both SMC2^ON^ and SMC2^OFF^ chromosomes.

A second possibility was that mitotic chromosomes from SMC2^OFF^ cells might be fragile, and the efficiency of their isolation might be markedly reduced. If true, this would mean that when conventional SILAC protocols are followed, and equal numbers of cells grown in heavy and light medium are mixed prior to chromosome isolation, the yield of all proteins, including core histones, would be reduced on mutant chromosomes. This is important, because if the recovery of all proteins is reduced because of a decreased chromosome yield, then specific differences because of the SMC mutations might be difficult to quantify. Importantly, efforts at correcting for potential losses during isolation by simply normalizing the data based on the amounts of histone H4 did not yield interpretable results.

The quantity of DNA correlates strongly with the amount of chromatin and number of chromosomes in a particular sample. Therefore, we isolated mitotic chromosomes from SMC2^ON^ and SMC2^OFF^ cells separately, and then used the DNA-binding dye PicoGreen to determine the relative amounts of DNA in both samples. We subsequently analyzed chromosome samples by mixing them in equal proportions, based on the DNA content, and subjected them to MS (supplemental Fig. S2*A*). When this method was used, chromosomes isolated from SMC2^ON^ and SMC2^OFF^ cells had comparable amounts of core histones. Finally, to determine the effects of the condition knockouts, the quantification of each protein of interest was normalized to that of histone H4. We could therefore perform a quantitative analysis of the nonhistone protein composition of chromosomes in the presence or absence of SMC2 (*i.e.* missing both condensin I and condensin II). All raw MS data in this study were deposited into the ProteomeXchange via The Proteomics ID Entifications (PRIDE) database (Data set identifier, PXD003427).

**Fig. 1.**
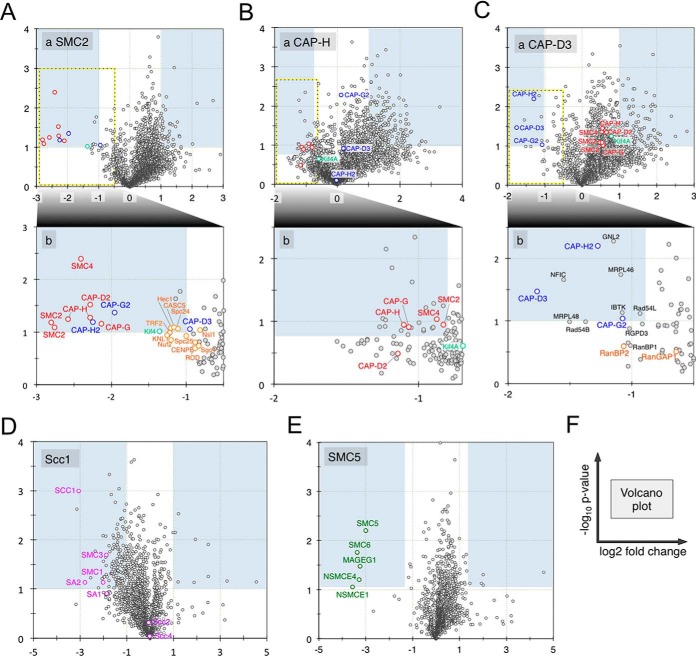
**Volcano plot for the relative quantitations performed by mass spectrometric analysis.** *A*, Relative changes in the protein composition in SMC2, (*B*) CAP-D3, (*C*) CAP-H, (*D*) Scc1, and (*E*) SMC5 knockout chicken DT40 cells. *F*, Explanation of the plot. Magnifications of the regions indicated in (A–C) are shown in the expanded plots below each panel.

**Fig. 2.**
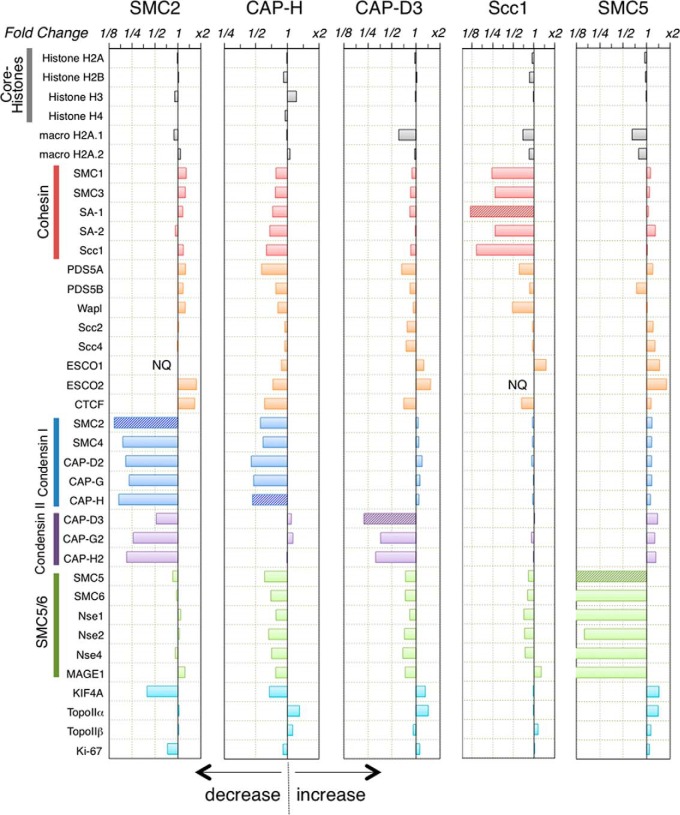
**Chromosomal association of individual SMC complexes.** The horizontal axis shows changes in protein abundance (on a logarithmic scale) in mitotic chromosomes depleted of individual proteins (SMC2, CAP-H, CAP-D3, Scc1, and SMC5) in DT40 cells. Chromosomal proteins with increased abundance on the depleted mitotic chromosomes are shown to the right of the vertical axis, and proteins with decreased abundance are shown to the left. The dependence of histone H1s and core histones with their variants, cohesin, condensin, SMC5/6 complex, and their related factors are shown. ND: not detected.

**Fig. 3.**
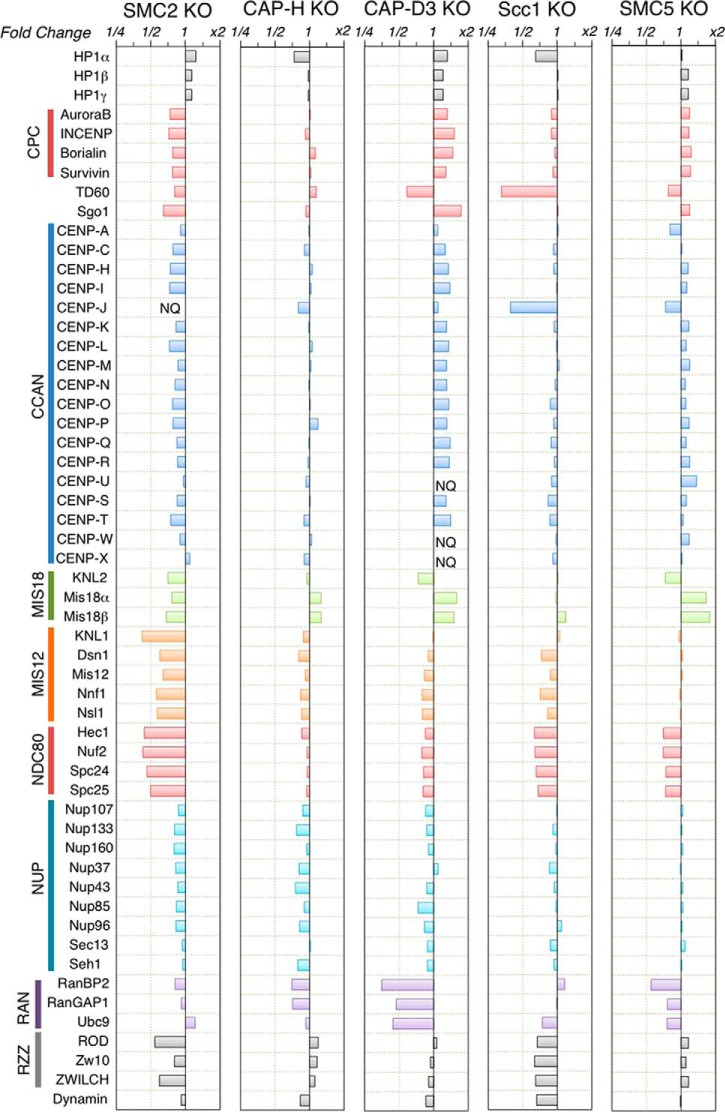
**Dependence of centromere proteins on SMC for chromosomal association.** The horizontal axis shows changes in protein abundances (on a logarithmic scale) in mitotic chromosomes depleted of individual proteins (SMC2, CAP-H, CAP-D3, Scc1, and SMC5) in DT40 cells. Complexes with increased abundance are shown to the right of the vertical axis, and complexes with decreased abundance are shown to the left. Complexes in the centromeric region are depicted with different colors.

The levels of most condensin I and condensin II subunits (SMC4, CAP-D2, CAP-G/G2, and CAP-H/H2) decreased by >75% in SMC2^OFF^ mitotic chromosomes. The sole exception was CAP-D3, whose levels fell by only 50%. The level of Kif4A, a chromosome-scaffold component, decreased to an extent similar to that observed for the condensin subunits. However, the levels of the abundant chromosome-scaffold component DNA topoisomerase II were unchanged between wild-type and SMC2^OFF^ chromosomes (Figs. 1 and 2). Loss of condensin also resulted in a marked decrease in the chromosomal association of KMN network (Knl-1/Mis12/Ndc80 complexes; Figs. 1*A* and 3). A slight decrease was also noted for several components of the constitutive centromere-associated network (CCAN) and for the Mis18 complex (Fig. 2). However, no significant change was observed in levels of the centromere-specific H3 variant CENP-A.

##### Dependences of Whole-chromosomal Proteomes on Condensin I or Condensin II

SMC2 depletion results in the loss of both condensin I and condensin II. Therefore, to separately examine the role of each subcomplex in determining the mitotic chromosomal proteome, we exploited conditional gene knockouts of CAP-H for condensin I and CAP-D3 for condensin II (DT40 CAP-H^ON/OFF^ or CAP-D3^ON/OFF^ cells) (39). Both forms of condensin are required for the structural integrity of mitotic chromosomes. Condensin I appears to play a more prominent role in lateral compaction, whereas condensin II seems to support axial compaction to a greater degree (14, 22, 23, 39).

The mitotic chromosomes of wild-type^heavy^ and CAP-H^OFF-light^(or CAP-D3^OFF-light^) cells were independently isolated and mixed to produce equal amounts of DNA, using PicoGreen standardization (supplemental Fig. S2*A*). Chromosomes depleted of CAP-H or CAP-D3 were found to contain 47 and 26% of the wild-type levels of CAP-H and CAP-D3, respectively. Overall, CAP-H-depleted chromosomes contained 46% of wild-type levels of condensin I and 106% of the wild-type levels of condensin II (Fig. 2). CAP-D3-depleted chromosomes contained 119% of the wild-type level of condensin I and 34% of the wild-type level of condensin II. Thus, our proteomic analyses of isolated mitotic chromosomes revealed that condensin I and condensin II were loaded independently onto mitotic chromosomes.

Our failure to completely deplete both complexes was because of technical limitations (supplemental Fig. S2*B*). We are only able to isolate mitotic chromosomes free of nuclei from cell populations with ≥80% mitotic cells. As a result, the degree of depletion obtained in various experiments depended upon the half-life of the targeted protein, in addition to the effects that its depletion has on the health of the culture. If we waited for longer periods of depletion for either CAP-H or CAP-D3, cultures became unhealthy (supplemental Figs. S2*C* and S2*D*) and we could no longer obtain a high enough mitotic index for mitotic chromosome isolation.

Similar to SMC2^OFF^, partial depletion of condensin I resulted in decreased KIF4A levels, but no large systemic changes in the chromosomal association of proteins from the main kinetochore subcomplexes (Fig. 2). Interestingly, we observed a marked decrease in levels of the RanBP2/RanGAP1 kinetochore subcomplexes upon the partial depletion of condensin II (Fig. 3).

##### Dependence of Whole-chromosomal Proteins on Cohesin

In addition to its function in holding sister chromatids together, cohesin was reported to be essential for the chromosomal loading of other proteins that are not associated with chromatid cohesion, but that organize into higher-order chromosome structures (50–52). For example, it was reported that condensin II abundance correlated with cohesin levels (14). In the present study, protein association with mitotic chromosomes was investigated in cells with or without the kleisin subunit Scc1/Rad21, a non-SMC subunit that bridges the SMC1 and SMC3 heads of cohesin (supplemental Fig. S2*E*; (41)). In Scc1^OFF^ chromosomes, all cohesin subunits (SMC1 (24%), SMC3 (27%), SA1 (27%), and SA2 (15%)) were significantly depleted from mitotic chromosomes (Figs. 1*D* and 2). In contrast, chromosomal loading of the cohesin-loading factors, Scc2 and Scc4, were unaffected (Figs. 1*E*, 1*D* and 2). Thus, Scc2 and Scc4 association with mitotic chromosomes was independent of cohesin. However, it has been reported that Scc2/4 loads coordinately with cohesin onto chromatin at centromeric and pericentromeric regions (53). If this is true, then our data suggest that centromeric and pericentromeric cohesin loading might be regulated by mechanisms that differ from bulk cohesin loading.

The loss of cohesin also resulted in decreased levels of chromosome-associated Wapl (48%) and PDS5A (61%). Wapl and PDS5A heterodimers were previously reported to bind SA1 or SA2 (54). However, Wapl and PDS5A levels did not decrease to the same extent as those of the core cohesin subunits. This finding suggested the existence of a cohesin-independent chromosome-loading pathway for Wapl and PDS5A, but suggested that they may require cohesin for stable association with chromosomes. The levels of PDS5B, another PDS5 homolog in vertebrates, were not significantly altered by the loss of cohesin, suggesting that PDS5B may be associated with a different biological pathway, possibly during interphase.

Cohesin depletion had no effect on the levels of chromosome-associated ESCO1. This enzyme acetylates SMC3 on chromosomes to stabilize cohesion, and deacetylation of SMC3 by HDAC8 promotes cohesin recycling (55). The cohesion-independent association of ESCO1 with chromosomes raises the possibility that ESCO1 might acetylate other chromosomal proteins in addition to SMC3. Finally, we noted that cohesin-depleted chromosomes had levels of condensin I, condensin II, and SMC5/6 complexes comparable to those observed on wild-type chromosomes (Fig. 2).

Functional interactions between cohesin and the DNA-replication machinery have been reported (10). The mini-chromosome maintenance (MCM) 2–7 helicase complex is an important component of the prereplication complex (pre-RC) that, upon association with Cdc45 and GINS complex to form the Cdc45/MCM/GINS complex, constitutes the active CMG DNA helicase that drives the elongation stages of DNA replication (56–60). Another pre-RC subcomplex, the origin-recognition complex (ORC), is stably bound to replication origins in the S phase.

**Fig. 4.**
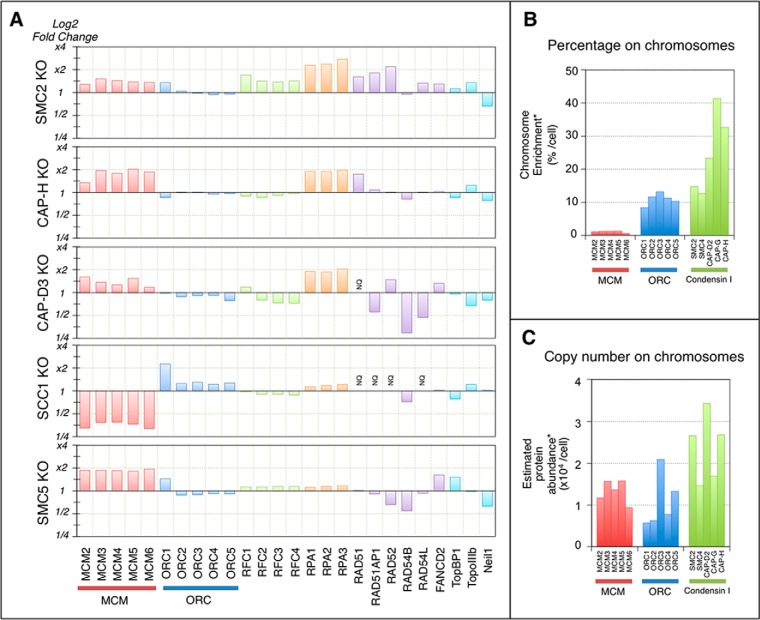
**Dependence of replication proteins on SMC for chromosomal association.** *A*, The vertical axis shows changes in protein abundance (on a logarithmic scale) in mitotic chromosomes individually depleted of various proteins (SMC2, Scc1, and SMC5) in DT40 cells. *B*, The fraction of proteins (by percentage) associated with mitotic chromosomes in DT40 cell extracts. *C*, The estimated copy number of proteins associated with mitotic chromosomes in a single mitotic DT40 cell.

MCM and ORC complexes were previously observed by our group on mitotic chromosomes, whereas others have reported their release from chromosomes during mitosis (61, 62). The proteomics approach employed here revealed that a small percentage of the MCM complex and ∼10% of the ORC remained associated with mitotic chromosomes in DT40 cells (Figs. 4*B* and 4*C*). Strikingly, the low level of the MCM complex remaining associated with mitotic chromosomes was further decreased in cohesin^OFF^ chromosomes, whereas the ORC levels increased slightly (Fig. 4*A*). Furthermore, the levels of the MCM complex were slightly increased in chromosomes depleted of condensin I, condensin II, and the SMC5/6 complex, whereas ORC levels were unchanged (Fig. 4*A*). These results suggested that cohesin enables a residual population of the MCM complex to remain associated with chromosomes during mitosis. The function, if any, of this complex is unknown.

##### Dependence of the Mitotic Chromosome Proteome on the SMC5/6 Complex

The SMC5/6 complex functions as a transcriptional regulator and in the removal of DNA linkages to allow sister chromatid segregation during mitosis (63). Moreover, this complex is recruited to support the repair of double-stranded DNA breaks, facilitating recombination between sister chromatids during meiosis by SUMOylating subunits of the cohesin complex (64, 65).

To assess the role of the SMC5/6 complex in mitotic chromosome assembly, SMC5-depleted chromosomes were obtained by growing cells in SILAC^light^ medium in the presence of doxycycline for 60 h prior to nocodazole blocking (SMC5^OFF^). Mitotic SMC5^ON^ and SMC5^OFF^ cells were mixed in equal numbers, followed by the isolation of mitotic chromosomes (supplemental Figs. S2*E* and S2*F*).

This analysis revealed 5 proteins that were highly dependent upon SMC5 for their associations with mitotic chromosomes, namely SMC6, NSMCE1, NSMCE2, MAGE1/NSMCE3, and NSMCE4 (Figs. 1*E* and 2). Quantitative analysis using SILAC peak ratios revealed that residual levels of these proteins in SMC5^OFF^ chromosomes were as follows: SMC5 (8.0%), SMC6 (9.9%), NSMCE1 (8.4%), NSMCE2 (6.3%), MAGE1/NSMCE3 (9.8%), and NSMCE4 (9.6%), as shown in Fig. 2. These data strongly suggested that these proteins all associated with chromosomes as components of SMC5/6 complexes.

With the exception of Rad54B, DNA-repair proteins showed no dependence upon SMC5/6 for chromosomal association (Fig. 4*A*). DNA damage activates a checkpoint response in G2 that blocks mitotic entry. Therefore, it is unlikely that high levels of active repair complexes would be associated with mitotic chromosomes under normal circumstances.

The loss of SMC5/6 was reported to result in abnormal chromatin structures and increased chromosome-segregation errors (28, 66), as well as defects in meiotic progression and the removal of sister chromatid links (32, 33). Although depletion of the SMC5/6 complex was accompanied by a slight reduction in chromosomal association of the Ndc80 complex, we observed no systemic changes in the chromosomal association of other centromere-/kinetochore-associated proteins or other proteins implicated in mitotic chromosome organization, DNA repair, recombination, or replication. Thus, our results suggested that the SMC5/6 complex does not play a major role in mitotic chromosome structure or dynamics.

##### Integration of Multiproteomics Data by nanoRF

Although each SILAC experiment yielded insight into the dependences of individual protein complexes on mitotic chromosomal structures and compositions, the results discussed thus far were focused on changes that were evident from direct inspection of the data. However, more subtle, but consistent relationships across the entire dataset cannot be readily distinguished by visual inspection alone. Among these could be subtle relationships between complexes, as well as proteins that functionally depend upon SMC complexes. In a previous study, the Random Forest (RF) machine-learning algorithm was used to distinguish functional chromosomal proteins from hitchhiker proteins in the whole proteome of mitotic chromosomes (35). Although it was assumed that a large number of training sets is required for RF analysis, we developed RFs using small training sets (*e.g.* protein complexes), referred to as “nanoRF” to distinguish this analysis from the previous RF approach. Using nanoRF, members of different complexes were found to belong to distinguishable cohorts. In contrast, proteins selected at random never formed a group (37).

Initially, we used nanoRF to determine whether our data set contained sufficient information to distinguish the formation of relevant chromosome-associated complexes from contaminants. Indeed, we successfully “fractionated” condensin (condensin I and/or condensin II), cohesin, SMC5/6, and the chromosome passenger complex (CPC) in silico, as shown by the statistical quality of the ROC curve and area-under-the-curve (AUC) analyses (supplemental Fig. S4).

**Fig. 5.**
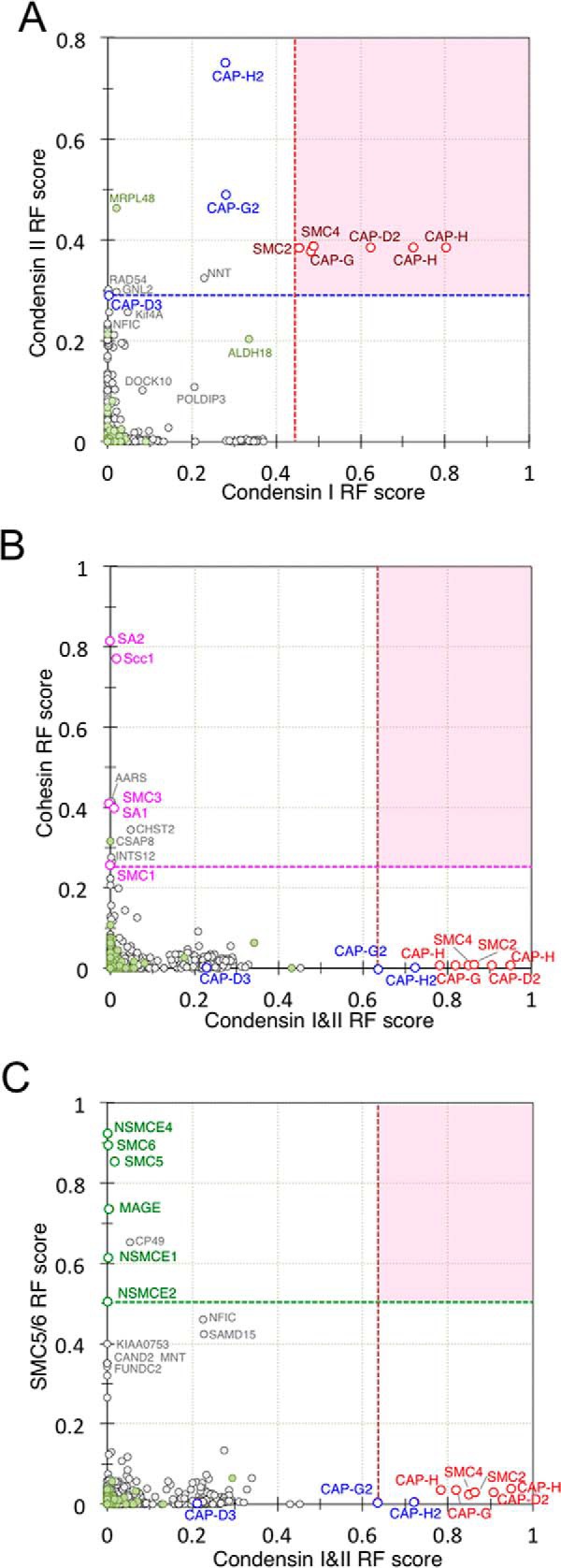
**NanoRF for SMC complexes.** Two-dimensional scatter graph plots of RF scores for (*A*) condensin I *versus* condensin II in nanoRF, (*B*) condensin *versus* cohesin, (*C*) and SMC5/6 *versus* condensin. Proteins with an adjusted RF score of >0.28 for condensin I and >0.3 for condensin II are shown within the red area (*A*). Proteins defined as “true” in the learning set for nanoRF analysis are colored: condensin, red; cohesin, purple; SMC5/6, orange; and cytoplasmic, green, with other significant proteins labeled accordingly.

To evaluate the contributions of condensin I and condensin II to chromosome structure, we first showed that nanoRF was capable of distinguishing between members of the condensin I and condensin II complexes by taking into account the interdependencies observed in various experiments (Fig. 5*A*). In this analysis, the only member that failed to fractionate as expected was CAP-D3, as discussed below. Surprisingly, KIF4A showed moderate co-fractionation with condensin II, but not with condensin I, despite evidence that KIF4A loading on mitotic chromosomes was dependent upon condensin I (Fig. 2). We speculate that Kif4A and condensin I cooperate to load onto mitotic chromosomes, but that Kif4A shows closer functional links with condensin II.

The functional association between condensin and cohesin complexes has been widely discussed, and recent *in vitro* experiments using *Xenopus* egg extracts (67) revealed that the loss of cohesin resulted in increased condensin II bound to chromatin. The nanoRF analysis clearly revealed a lack of evidence for functional associations between condensin and cohesin (Fig. 5*B*) or between condensin and SMC5/6 (Fig. 5*C*). These findings corroborated our bulk proteomics data (Figs. 1 and 2), indicating that despite their apparent structural similarities, the three SMC complexes are functionally independent on mitotic chromosomes.

**Fig. 6.**
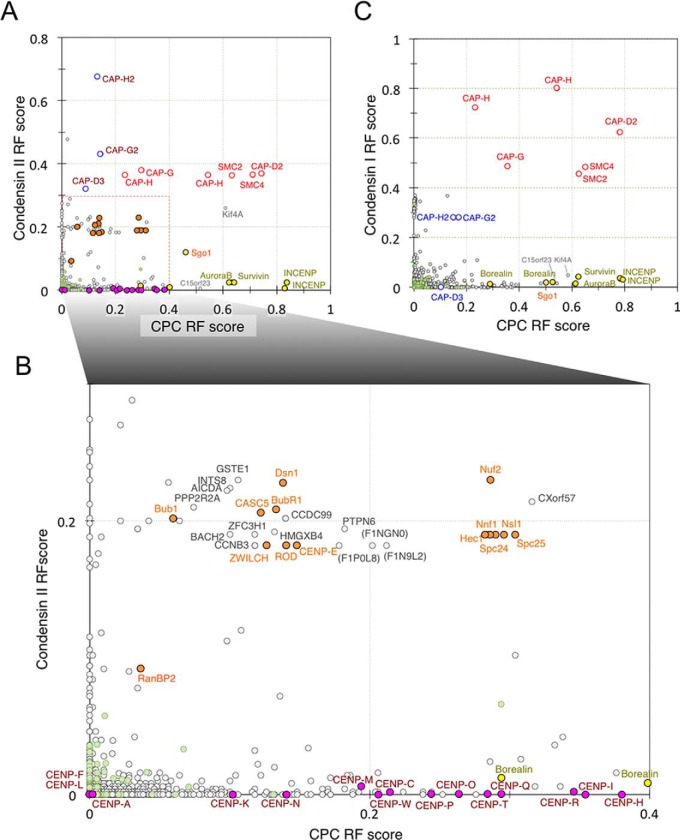
**nanoRF for condensin I and condensin II.** Two-dimensional scatter graph plots of RF scores for (*A*) CPC *versus* condensin II and (*B*) CPC *versus* condensin I. *C*, Magnification of the region indicated in (A). Key proteins are color coded by category: condensin, red; CPC, yellow; kinetochore, orange; and CCAN, pink.

We next used nanoRF to determine whether the CPC showed a specific dependence upon condensin I or condensin II. Indeed, CPC fractionation yielded high values for condensin I and KIF4A, but low values for condensin II (Figs. 6*A* and 6*B*), suggesting a specific relationship between condensin I and the CPC. However, the reciprocal fractionation of condensins yielded near-zero values for the CPC (Figs. 6*A* and 6*B*). We interpret these results to suggest that the CPC associates with chromosomes upstream of condensin I function, such that the CPC might regulate condensin I and only indirectly regulate condensin II. NanoRF analysis also suggested weak interdependencies of the CCAN, NDC80, and RZZ complexes with the CPC (Figs. 6*A* and 6*B*), as well as weak interdependencies of the NDC80 and RZZ complexes, but not the CCAN complex, with condensin II (Figs. 6*A* and 6*B*).

Taken together, this analysis suggested that the CPC influences the structure of the entire centromere/kinetochore, whereas condensin II may interact predominantly with the outer kinetochore.

##### Clustering Multiple nanoRFs Against 12 Functional Complexes on Mitotic Chromosomes

NanoRF enables the prediction of functional relationships that occur with any target complex, including those for which no genetic knockout has yet been analyzed. The 6 independent nanoRF analyses mentioned previously were validated for condensin, condensin I, condensin II, cohesin, SMC5/6, and the CPC. We also performed 6 additional independent nanoRF analyses for histones, CCAN and Nup-Ran complexes, the kinetochore (Mis12, Ndc80, and RZZ complexes), chromosomal scaffolds, and ribosomal proteins. These yielded favorable ROC curves, implying successful separation quality (supplemental Fig. S4*B*). In contrast, ROC analysis showed poor-quality fractionation for histone H1s and telomere proteins, implying that our datasets were not informative for these elements (supplemental Fig. S4*C*).

Given that nanoRF analysis generates values for all proteins under consideration, the 12 sets of nanoRF data represented in this study could be used to construct heat maps, with the 5,058 proteins quantified in at least 1 experiment classified into 8 branches (supplemental Fig. S5). Of these branches, 4833 proteins were classified into a single majority branch, and another 2 branches combined included 112 ribosomal proteins (supplemental Fig. S5). The major branch represents proteins with no apparent functional relationship with the genes targeted or complexes analyzed in our studies. Although the ribosomal proteins could be linked with chromosome structure via their association with the mitotic chromosome periphery (68), they did not exhibit obvious links with the specific complexes analyzed above, and we did not perform nanoRF analysis for the poorly-defined chromosome periphery compartment. Interestingly, each of the other six branches had a unique composition.

**Fig. 7.**
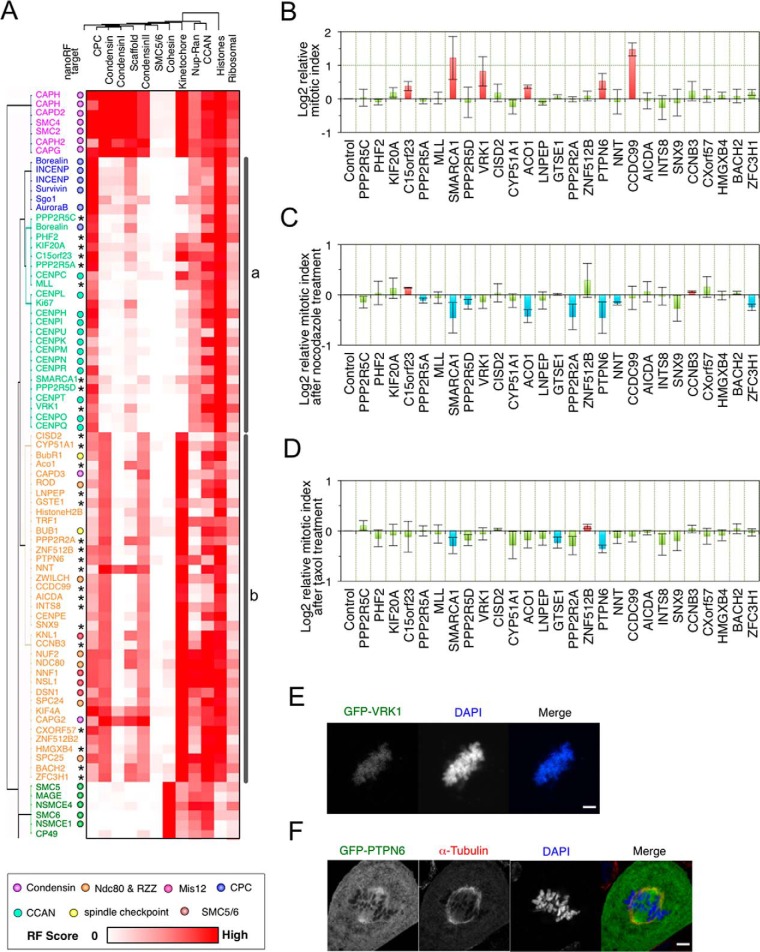
**Cluster analysis with multiple nanoRF.** *A*, Heat map depicting the combined results of 5,058 proteins from nanoRF targeting condensin, condensin I, condensin II, cohesin, SMC5/6, CPC, histones, CCAN, Nup-Ran complexes, kinetochore, chromosomal scaffolds, and ribosomal proteins. Magnification of the region is indicated in supplemental Fig. S5. The 2 branch classes (a, b) are shown on the right. * shows the siRNA target in (*B–D*). *B*, Relative mitotic index of HeLa cells after 28 individual siRNA treatments against human orthologs and (*C*, *D*) after siRNA and nocodazole (*C*) or taxol (*D*) treatment. siRNA targets are shown on the left. Red and blue bars indicate statistical increases and decreases in the mitotic index, respectively. U2OS cells transiently expressing Halo-VRK1 (*E*) or GFP-PTPN6 (*F*). Cells were stained for α-tubulin (red, *F*) and DNA (blue; *E*, *F*).

Condensin subunits, with the exception of CAP-D3, were included in a branch that also included kinetochore proteins from the NDC80, MIS12, and RZZ complexes (73 proteins; Fig. 7*A*). Collectively, our proteomics data suggested that CAP-D3 undergoes functional associations that are distinct from other condensin subunits. This could be explained in two ways. CAP-D3 might be replaced by another factor in the condensin II complex. This is unlikely, however, as CAP-D3 appears to be an authentic member of condensin II. The levels of other condensin II-specific subunits, CAP-G2 and CAP-H2, were strongly reduced in CAP-D3-depleted chromosomes (Fig. 2). The fact that CAP-D3 showed a lower extent of dependence on SMC2 relative to other condensin II subunits suggested that CAP-D3 might have an additional capacity to bind chromosomes independent of the rest of the condensin II complex.

The CPC, CCAN, and SMC5/6 complexes displayed functional clustering with several unexpected proteins (Fig. 7*A*), including proteins that were uncharacterized at the time the chicken protein database used for these analyses was assembled (35). Most subunits of the NDC80 and Mis12 complexes were included in 1 branch, with certain proteins in neighboring branches also functioning in the capture of microtubules at kinetochores (Fig. 7*A*).

##### Validation of Centromere-/Kinetochore-associated Candidates from Multiple-nanoRF Analysis, Using siRNAs

Cluster-analysis scores obtained using multiple nanoRFs showed ∼30 unexpected and/or novel proteins in branches containing condensin subunits and centromere proteins. Therefore, we attempted to verify whether nanoRF prediction could identify novel proteins that affect mitotic progression. The human orthologs of 27 candidate chicken genes were identified and subjected to siRNA-mediated knockdown in human HeLa cells (supplemental Table S3). We then determined the mitotic index in the respective knockdown cells by flow cytometry with PI labeling and histone H3 phospho-S10 staining. We observed a significantly higher mitotic index (compared with controls) following silencing of C15orf23, SWI/SNF-related, matrix-associated, actin-dependent regulator of chromatin, subfamily A, member 1 (SMARCA1), vaccinia-related kinase 1 (VRK1), the iron-responsive element-binding protein, aconitase 1 (ACO1), tyrosine-protein phosphatase nonreceptor type 6 (PTPN6), and coiled-coil domain-containing protein 99 (CCDC99) (Fig. 7*B*, supplemental Table S2).

Functional analyses by other groups have linked CCDC99 and C15orf23 to kinetochores (69, 70), corroborating some of our results and suggesting that our nanoRF analysis did indeed reveal additional, novel proteins of interest. Importantly, those analyses were published after construction of the database used in the present analysis (35). Thus, CCDC99 was identified as a kinetochore component that interacts with the RZZ complex and was named Spindly (71–73). This finding is consistent with our multiple nanoRF clustering results, wherein CCDC99 was adjacent to ZWILCH and in the same branch as ROD (Fig. 7*A*). In addition, C15orf23 was also later characterized and renamed as the *s*mall *k*inetochore-*a*ssociated *p*rotein (SKAP) (74, 75). Gene-expression analysis in adenocarcinomas based on microarray data showed that VRK1 was correlated with members of the mitotic cell-cycle gene network, including CENP-A, CENP-K, CENP-N, CENP-Q, Bub1, BubR1, SMC2, and CAP-G (76). Moreover, these data showed that the loss of VRK1 led to *K_i_*-67 mislocalization and decreased cell viability associated with a polyadenosine diphosphate ribose polymerase inhibitor (76). These findings are also consistent with our multiple nanoRF results, wherein VRK1 was in same branch as CENP-K, CENP-N, CENP-Q, and *K_i_*-67 (Fig. 7*A*). Furthermore, a Halo-tagged VRK1 variant exhibited chromosomal localization during mitosis in human U2OS cells (Fig. 7*E*).

We next considered the possibility that some of the proteins linked to mitotic chromosomes by nanoRF analysis might also be linked to the *s*pindle *a*ssembly *c*heckpoint (SAC). In such a case, the mitotic indices of knockdown cells would be expected to be lower relative to controls after nocodazole treatment, because of SAC inactivation. As shown in Fig. 7*C* and supplemental Table S2, the knockdown of SMARCA1, ACO1, PTPN6, and serine/threonine-*p*rotein *p*hosphatase *2A r*egulatory subunit B *a*lpha isoform (PPP2R2A) all showed lower mitotic indices compared with controls after nocodazole treatment (Fig. 7*C*, supplemental Table S2). Moreover, the knockdown of SMARCA1 and PTPN6 resulted in significantly lower mitotic indexes after taxol treatment that disturbs microtubule de-polymerization (Fig. 7*D*, supplemental Table S2). These data suggested that these genes might be linked to the SAC-activation pathway. Our multiple-nanoRF analysis revealed that ACO1, PTPN6, and PPP2R2A were in same branch as the SAC components Bub1 and BubR1. Consistent with these analyses, a green-fluorescent protein (GFP)-PTPN6 fusion protein localized to spindles during mitosis in human U2OS cells (Fig. 7*F*). Although more experiments are required to clearly establish the roles of these proteins in mitosis, our analyses suggested that nanoRF analysis is a useful tool for detecting functional interactions between known and novel proteins.

## DISCUSSION

Results presented in this study demonstrated that the condensin, cohesin, and SMC5/6 SMC complexes associate with mitotic chromosomes independently of each other. We also provide evidence that cohesin and SMC5/6 levels were not altered in mitotic chromosomes that were simultaneously depleted of both condensin I and condensin II, using SMC2^OFF^ cells.

NanoRF analysis of the proteomics results shown here revealed that, of the 5058 proteins observed in this study, only 113 (one-third of which were well-known centromere/kinetochore proteins) behaved as though they were functionally linked to components important for chromosomal structures or segregation. This number may be an underestimate, as we did not attempt to specifically analyze the chromosome periphery compartment. However, because the bulk of the periphery compartment appeared to be composed of nucleolar components, it was possible that the nanoRF branch containing 112 ribosome-associated proteins did, in fact, correspond to the chromosome periphery. Even if that were true and the number of specific chromosome-associated proteins rose to 225, it is clear that the vast majority of proteins associated with mitotic chromosomes are evidently “hitchhikers” that do not support chromosome structure or segregation.

**Fig. 8.**
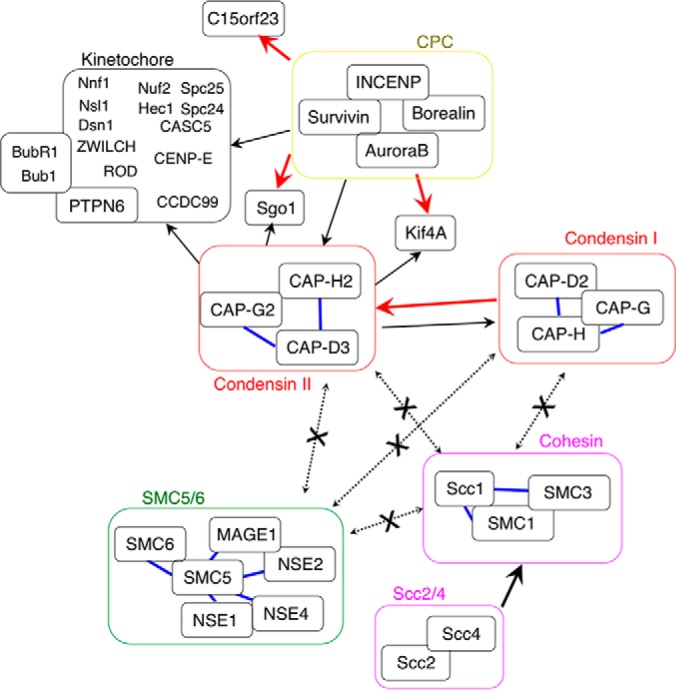
**A schematic model of the functional interactions occurring on mitotic chromosomes, identified in this study.** Regulation suggested by the nano-RF results is indicated by red (RF score > MCC) or black (MCC > RF score) arrows. Blue lines indicate strong interactions found in this proteomics study. Dotted lines indicate a lack of statistical support for interaction.

Partial depletion of the condensin I subunit CAP-H resulted in a slight decrease in KIF4A association with mitotic chromosomes. In a reciprocal experiment, KIF4A depletion resulted in the loss of all condensin I subunits (21). These results suggested that condensin I and KIF4A are mutually interdependent for association with mitotic chromosomes (Fig. 8). In contrast, partial depletion of the condensin II subunit CAP-D3 had no effect on KIF4A levels, suggesting that condensin II and KIF4 function are not tightly linked. Significant differences in topoisomerase IIα levels were not detected in the presence or absence of SMC2. This agreed with a previous biochemical study performed using SMC2-knockout chromosomes (19). However, a slight increase in topoisomerase IIα was observed in CAP-H- or CAP-D3-knockout chromosomes. We speculate that the loss of condensin I or condensin II alone may cause relatively mild perturbations of chromosome structures during mitosis, and these may result in increased levels of topoisomerase IIα association possibly as an adaptive compensatory response (Fig. 8).

Genome-wide mapping through ChIP-seq and immunofluorescence studies have shown that SMC2 and condensin I (using CAP-H) are both highly enriched at the centromeres of chicken DT40 cells during mitosis (77). Here, we showed that the simultaneous loss of both condensin complexes in SMC2-KO cells resulted in a significant depletion of members of the KMN network, with much milder effects on the CCAN and the CPC. These changes were not evident following partial depletion of either condensin I or condensin II alone, although the latter resulted in marked changes in the chromosomal levels of the RanBP2/RanGAP1 subcomplex. It is possible that the lesser extent of depletion observed for the condensin I and condensin II complexes was not sufficient to disrupt the kinetochore structure. Alternatively, both complexes may be to some extent redundant in their ability to create a chromosomal architecture conducive to kinetochore assembly.

Cohesin not only mediates sister chromatid cohesion during mitosis, but also contributes to gene expression in interphase, presumably by influencing chromosomal loop structures (78). Recent findings demonstrated that CCCTC-binding factor (CTCF) and cohesin colocalize on chromosomes (50–52). Cohesin is thought to be recruited to specific genomic sites via association with CTCF, and this association is proposed to promote the formation of chromatin loops (79). Our data showed that a proper balance of condensin I and II is also required to stabilize the chromosomal association of CTCF, suggesting that condensin might also be required for CTCF to modify chromosome structures.

Interestingly, our nanoRF analysis suggested that condensin II, but not condensin I, may exhibit functional interactions with the NDC80 and RZZ complexes. However, decreased levels of the NDC80 complex were observed only after depletion of both condensin I and condensin II. It is possible that, although loading of the NDC80 complex onto mitotic chromosomes is primarily linked to condensin II, condensin I may compensate at least in part for the loss of condensin II during an adaptive response. A similar mechanism may explain why nanoRF suggested a greater functional interaction of KIF4A with condensin II as compared with condensin I, whereas we observed that association of KIF4A with mitotic chromosomes decreased in cells harboring CAP-H, but not CAP-D3, knockouts, and that both condensin I and condensin II subunits were reduced on KIF4A-depleted chromosomes (21).

When we integrated multiple nanoRF analyses by clustering, we observed that some proteins only “fractionated” in silico in their specific nanoRF, whereas others fractionated in the nanoRFs of multiple complexes. This multiple-nanoRF analysis predicted some novel kinetochore-associated proteins, two of which have been confirmed in experiments from other groups (69, 74). Most subunits of the NDC80 and Mis12 complexes were included in one branch, with neighboring branches containing other proteins thought to function in capturing microtubules at kinetochores. Taken together, our data indicate that nanoRF analysis identified “signatures” of kinetochore microtubule-binding proteins, based on their dependence on SMC components of the chromosome scaffold.

Of the proteins shown by nanoRF analysis to link to kinetochores, SKAP has been reported to interact with the CENP-E and KMN networks (74, 80, 81). Interestingly, nanoRF clustering predicted functional interactions between SKAP and CENP-C. CENP-C interacts with the Mis12 complex and is known to form a platform linking the inner and outer kinetochores. Thus, nanoRF analysis suggested further avenues of exploration in analyzing CENP-C functions.

Of the proteins linked to kinetochore function by nanoRF, several showed no change in the mitotic index after siRNA-mediated knockdown. Although the siRNAs employed may have been ineffective in target mRNA silencing, this consideration does not rule out the possibility that these proteins displayed meaningful functional links with other kinetochore proteins. Indeed, 5 of the nanoRF candidates showed an apparent weakening of the SAC when their depletion was combined with nocodazole treatment. All of these candidates (except for SMARCA1) clustered near BubR1 and Bub1 in our multiple-nanoRF analysis. Indeed, GFP-tagging and localization tracking revealed that 1 of them, PTPN6 whose depletion led to weakening of the SAC with stabilized microtubules following taxol treatment, can bind to spindles during mitosis.

### 

#### 

##### Perspectives

Given that most proteins involved in mitotic chromosome architecture, as well as kinetochore structure and function, have likely been identified, it is critical to develop novel orthogonal methods to look for further functional relationships. This has traditionally been achieved by yeast 2-hybrid analysis (82), analysis of protein-immunoprecipitation results (83–87), analysis of protein co-localization (70), analysis of protein proximity (88), and multiclassifier combinatorial proteomics (35). NanoRF offers an additional approach to discover subtle functional associations in proteomics data. The integration of various combinations of these methods in the future should provide significant advances in predicting the functions of novel proteins, as well as novel functions for known proteins.

## Supplementary Material

Supplemental Data

## Footnotes

^1^ The abbreviations used are:

SMC: structural maintenance of chromosomes
ACO1: aconitase 1
ADP: adenosine diphosphate
ATP: adenosine triphosphate
AUC: area under the curve
BSA: bovine serum albumin
CCAN: constitutive centromere-associated network
CCDC99: coiled-coil domain-containing protein 99
CPC: chromosome passenger complex
DMEM: Dulbecco's Modified Eagle's Medium
FBS: fetal bovine serum
GFP: green-fluorescent protein
KMN: Knl-1/Mis12/Ndc80 complexes
MCC: Matthews correlation coefficient
MCM: mini-chromosome maintenance
MS: mass spectrometry
ORC: origin-recognition complex
PBS: phosphate-buffered saline
PI: propidium iodide
PPP2R2A: serine/threonine-protein phosphatase 2A, regulatory subunit B, alpha isoform
ppm: parts per million
pre-RC: prereplication complex
PTPN6: tyrosine-protein phosphatase nonreceptor type 6
RF: Random Forest
ROC: receiver operating characteristic
RPMI: Roswell Park Memorial Institute
SAC: spindle assembly checkpoint
SCX: strong cation-exchange chromatography
SDS-PAGE: sodium dodecyl sulfate-polyacrylamide gel electrophoresis
SILAC: stable isotope labeling with amino acids in cell culture
siRNA: small interfering RNA
SKAP: small kinetochore-associated protein
SMARCA1: SWI/SNF-related, matrix-associated, actin-dependent regulator of chromatin, subfamily A, member 1
VRK1: vaccinia-related kinase 1.
