# Supplementary material for: Proteomics Analysis with a Nano Random Forest Approach Reveals Novel Functional Interactions Regulated by SMC Complexes on Mitotic Chromosomes
Source: Mol Cell Proteomics. 2016 May 26;15(8):2802–18. doi: 10.1074/mcp.M116.057885 (PMC4974353; doi:10.1074/mcp.M116.057885)
Supplement: Supplemental Data [file supp_15_8_2802__index.html]

Proteomics analysis with a nano Random Forest approach reveals novel functional interactions regulated by SMC complexes on mitotic chromosomes — Proteomics Analysis with a Nano Random Forest Approach Reveals Novel Functional Interactions Regulated by SMC Complexes on Mitotic Chromosomes — Mitotic Chromatin Proteomics with Nano Random Forest — Supplemental Data 

# Proteomics Analysis with a Nano Random Forest Approach Reveals Novel Functional Interactions Regulated by SMC Complexes on Mitotic Chromosomes

## Supplemental Data

- Fig.S1-S5 and Table 2 and 3. (.pdf, 650 KB) - Supplemental information
- Table S1 (.txt, 3.9 MB) - Table S1
