# Supplementary material for: Proteomics Analysis with a Nano Random Forest Approach Reveals Novel Functional Interactions Regulated by SMC Complexes on Mitotic Chromosomes
Source: Mol Cell Proteomics. 2016 May 26;15(8):2802–18. doi: 10.1074/mcp.M116.057885 (PMC4974353; doi:10.1074/mcp.M116.057885)
Supplement: Supplemental Data [file 10.1074_M116.057885_mcp.M116.057885-1.pdf]

Supplemental Information

Figure S1. Nucleosome-spacing assay of isolated wild-type and SMC2^{OFF} chromosomes. The assay was performed in the presence of ~3 µg (OD_{260} = 0.06) of chromosomal DNA. Wild-type chromosome containing 0, 0.5, 1, 2, or 5 µg micrococcal nuclease (lanes 1–5). SMC2^{OFF} chromosome containing 0, 0.5, 1, 2, or 5 µg micrococcal nuclease (lanes 6–10)

Figure S2. (A, E) Experimental design of proteomics analysis using (A) SMC2, CAP-H, or CAP-D3 knockout cells and (E) Scc1 or SMC5 CAP-D3 knockout cells. (B) SDS-PAGE gel stained with Coomassie brilliant blue and immunoblot analyses of the proteins targeted for depletion in isolated mitotic chromosomes from SMC2^{OFF} or CAP-D3^{OFF} cells. (C, D, F) Growth curves of CAP-G^{OFF} (C), CAP-D3^{OFF} (D), or SMC5^{OFF} (F) cells. The cell types are labeled in the left box (Wild type, -Doxycycline (ON), or +Doxycycline (OFF)).

Figure S3. Comparison between the results from biological replicates in SMC2 (A), CAP-H (B), CAP-D3 (C), Scc1 (D), and SMC5 (E) KO cells. The red number for each comparison indicates the Pearson correlation coefficient.

Figure S4. The receiver operating characteristic (ROC) curves and the area under the ROC curve (AUC) of all nanoRF analyses in this study. The AUC values indicated in bold font are over 0.8.

Figure S5. Entire heat map, shown in Fig. 7A, depicting the combined results of 5,058 proteins from nanoRF targeting condensin, condensin I, condensin II, cohesin, SMC5/6, CPC, histones, CCAN, Nup-Ran complexes, kinetochore, chromosomal scaffolds, and ribosomal proteins. The 8 classified branches are indicated with different colors, and the branches are separated into 4 classes (a–d) on the right.

Table S1. List of all identified proteins

Table S2. Mitotic index measurement of siRNA knockdown in HeLa cells.
Log2 mitotic index (MI), standard deviation (SD), and p-value (p) are shown. Bold numbers indicate p values that are <0.05.

Table S3. The orthologs of knockdown targets and level of amino acid-sequence homology between the chicken and human proteins

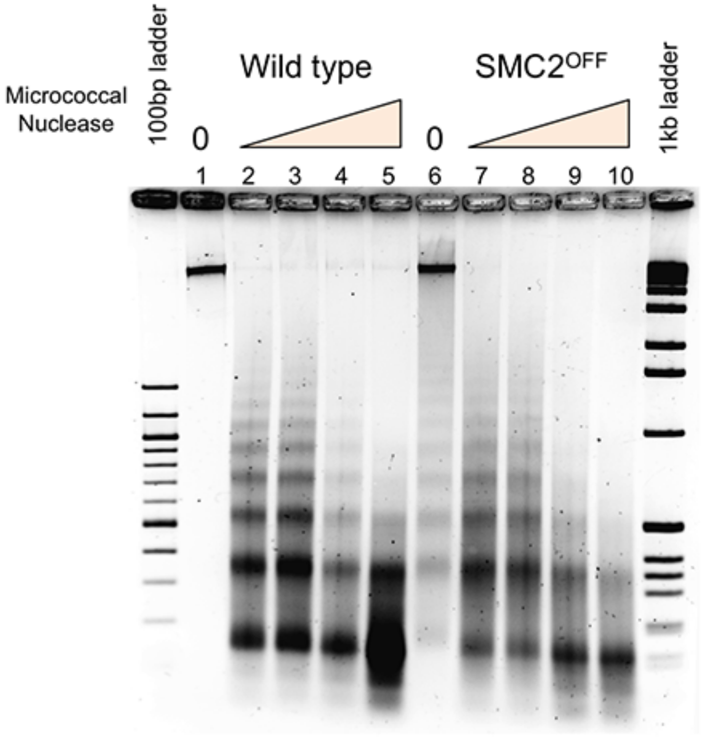
Micrococcal Nuclease
100bp ladder
Wild type
SMC2^OFF
1kb ladder
0
0
1 2 3 4 5 6 7 8 9 10

Fig. S1

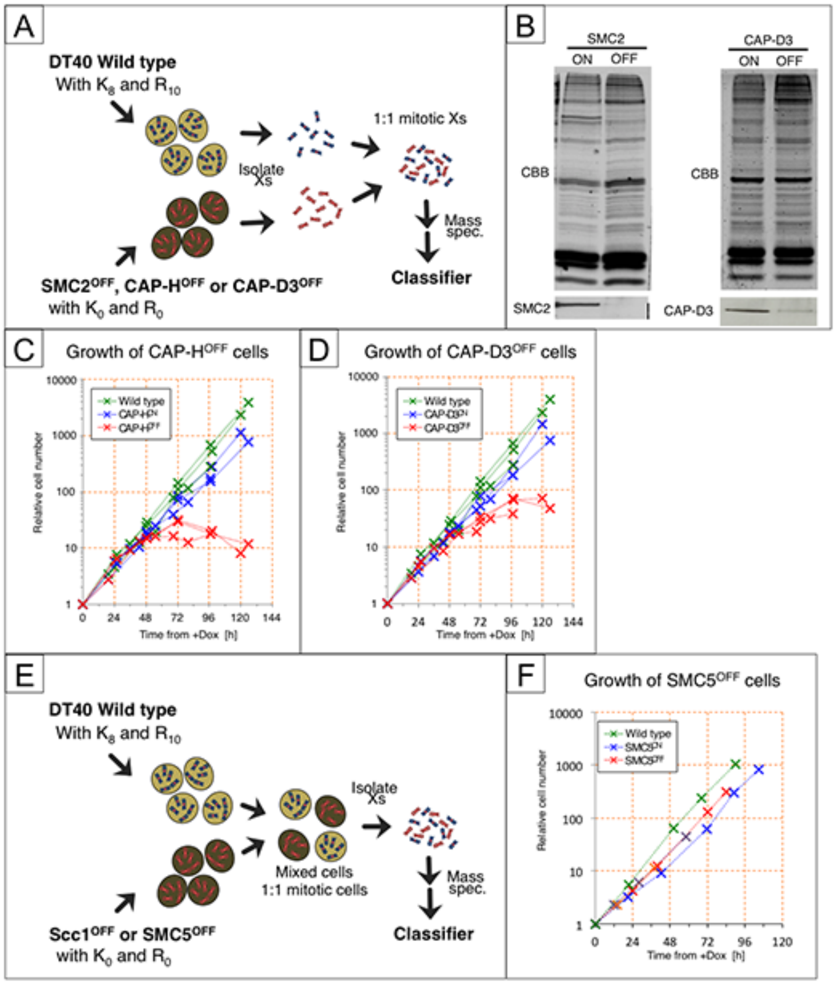
A
DT40 Wild type
With K_8 and R_{10}
Isolate Xs
1:1 mitotic Xs
Mass spec.
Classifier
SMC2OFF, CAP-HOFF or CAP-D3OFF
with K_0 and R_0
B
SMC2
ON OFF
CBB
SMC2
CAP-D3
ON OFF
CBB
CAP-D3
C
Growth of CAP-HOFF cells
D
Growth of CAP-D3OFF cells
Relative cell number
Time from +Dox [h]
E
DT40 Wild type
With K_8 and R_{10}
Isolate Xs
Mixed cells
1:1 mitotic cells
Mass spec.
Classifier
Scc1OFF or SMC5OFF
with K_0 and R_0
F
Growth of SMC5OFF cells
Wild type
Relative cell number
Time from +Dox [h]

Fig. S2

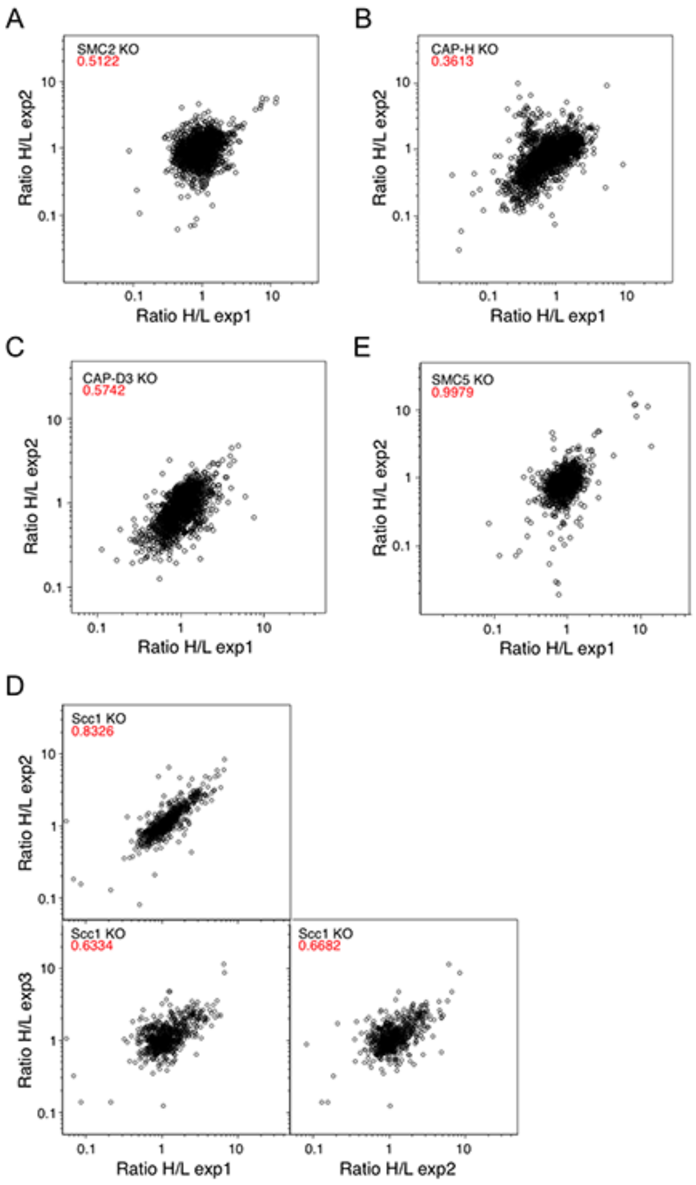
A
SMC2 KO
0.5122
Ratio H/L exp2
Ratio H/L exp1
B
CAP-H KO
0.3613
Ratio H/L exp2
Ratio H/L exp1
C
CAP-D3 KO
0.5742
Ratio H/L exp2
Ratio H/L exp1
E
SMC5 KO
0.9979
Ratio H/L exp2
Ratio H/L exp1
D
Scc1 KO
0.8326
Ratio H/L exp2
Scc1 KO
0.6334
Ratio H/L exp3
Ratio H/L exp1
Scc1 KO
0.6682
Ratio H/L exp2

Fig. S3

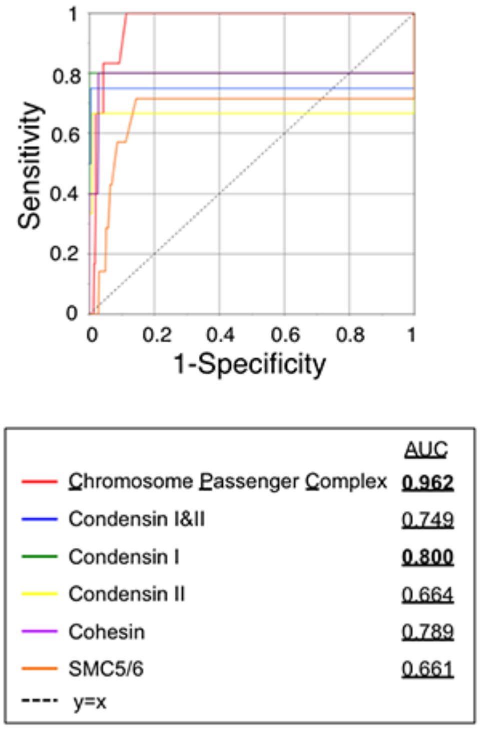
Sensitivity
1-Specificity
0
0.2
0.4
0.6
0.8
1
AUC
Chromosome Passenger Complex 0.962
Condensin I&II 0.749
Condensin I 0.800
Condensin II 0.664
Cohesin 0.789
SMC5/6 0.661
y=x

Fig. S4

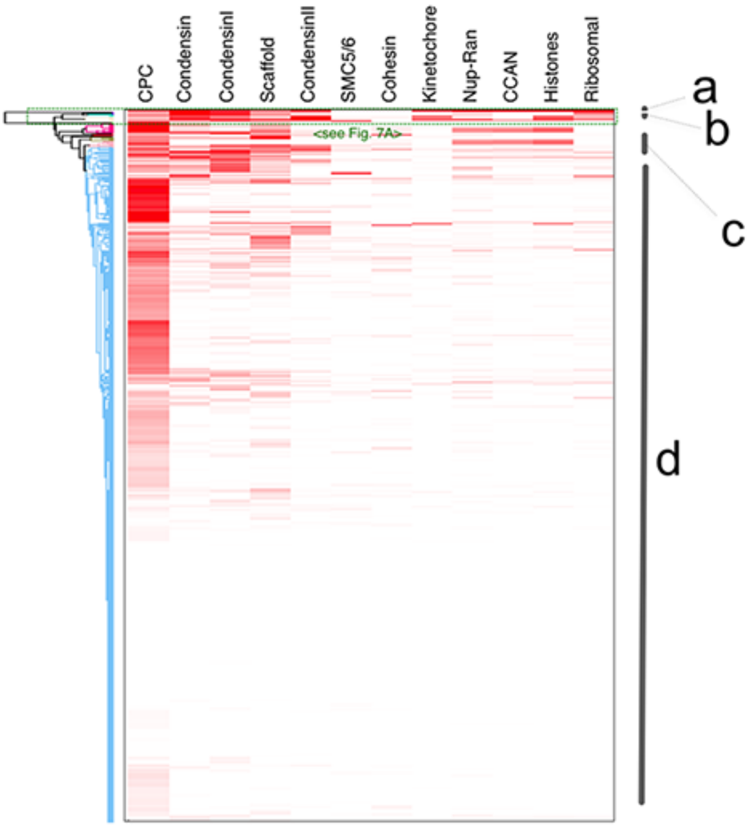
CPC
Condensin
CondensinI
Scaffold
CondensinII
SMC5/6
Cohesin
Kinetochore
Nup-Ran
CCAN
Histones
Ribosomal
<see Fig. 7A>
a
b
c
d

Fig. S5

Table S2 Mitotic index measurement of siRNA knockdown in HeLa cells.

Log2 mitotic index (MI), standard deviation (SD), and p-value (p) are shown. Bold number indicate < 0.05 in p-value.

	No treatment			Nocodazole treatment			Taxol treatment		
	MI	SD	p value	MI	SD	p value	MI	SD	p value
Control	0	0	-	0	0	-	0	0	-
PPP2R5C	0.03	0.25	0.847	-0.14	0.12	0.165	0.10	0.10	0.208
PHF2	-0.10	0.09	0.190	0.04	0.23	0.764	-0.15	0.17	0.258
KIF20A	0.18	0.14	0.156	0.13	0.20	0.385	-0.08	0.22	0.579
C15orf23	0.38	0.14	**0.042**	0.14	0.01	**0.003**	-0.11	0.31	0.588
PPP2R5A	-0.07	0.08	0.236	-0.11	0.05	0.068	0.00	0.10	0.984
MLL	0.02	0.17	0.882	-0.06	0.12	0.505	-0.06	0.18	0.613
SMARCA1	1.22	0.64	0.081	-0.46	0.30	**0.042**	-0.29	0.16	0.089
PPP2R5D	-0.11	0.46	0.724	-0.19	0.10	0.072	-0.18	0.11	0.102
VRK1	0.82	0.44	0.084	-0.14	0.13	0.215	-0.06	0.12	0.485
CISD2	0.17	0.26	0.378	0.04	0.18	0.725	0.03	0.02	0.121
CYP51A1	-0.24	0.22	0.195	-0.12	0.13	0.266	-0.28	0.27	0.214
ACO1	0.36	0.06	**0.009**	-0.42	0.13	**0.023**	-0.18	0.16	0.204
LNPEP	-0.14	0.05	**0.047**	-0.11	0.17	0.386	-0.15	0.13	0.194
GTSE1	0.06	0.06	0.249	0.01	0.02	0.468	-0.23	0.11	0.068
PPP2R2A	-0.02	0.08	0.730	-0.44	0.25	**0.033**	-0.29	0.18	0.106
ZNF512B	0.08	0.15	0.423	0.29	0.34	0.303	0.09	0.04	0.066
PTPN6	0.52	0.23	0.061	-0.45	0.31	**0.040**	-0.36	0.08	**0.015**
NNT	-0.09	0.36	0.709	-0.17	0.03	0.008	-0.13	0.12	0.201
CCDC99	1.47	0.19	**0.006**	-0.06	0.23	0.754	-0.11	0.11	0.234
AICDA	-0.06	0.24	0.696	0.06	0.20	0.625	-0.04	0.04	0.287
INTS8	-0.26	0.36	0.333	-0.03	0.18	0.849	-0.27	0.21	0.157
SNX9	-0.12	0.40	0.655	-0.27	0.26	0.185	-0.20	0.20	0.221
CCNB3	0.23	0.29	0.306	0.07	0.02	**0.023**	0.04	0.08	0.504
CXorf57	0.09	0.19	0.514	0.16	0.21	0.305	-0.11	0.16	0.366
HMGXB4	0.10	0.10	0.233	-0.02	0.08	0.800	-0.09	0.11	0.292
BACH2	0.08	0.21	0.582	0.04	0.03	0.129	0.04	0.10	0.546
ZFC3H1	0.19	0.09	0.066	-0.25	0.07	**0.020**	-0.03	0.07	0.488
RanBP2	-0.11	0.09	0.160	-0.12	0.12	0.400	-0.30	0.16	0.160
Ska3	3.80	1.12	0.028	0.37	0.02	**0.001**	0.21	0.04	**0.014**
Mad2	-1.20	0.69	0.096	-1.51	0.22	**0.002**	-1.44	0.57	**0.048**

Table S3 The orthologues for knockdown targets and level of amino-acids sequential homology between chicken and human

Acc (Chicken)	Acc (Human)	Gene Symbol	Gene Acc (Human)	Homology dentity (%)
Q5F3C6	Q13362	PPP2R5C	NM_178588	94.847
F1NVY2	O75151	PHF2	NM_005392	79.473
E1C5A3	Q15172	PPP2R5A	NM_006243	91.224
F1NET5	Q03164	MLL	NM_005933	72.142
F1P3Q4	P28370	SMARCA1	NM_139035	85.327
F1P090	Q14738	PPP2R5D	NM_006245	89.448
F1NDK1	Q99986	VRK1	NM_003384	69.082
R4GLS1	Q8N5K1	CISD2	NM_001008388	94.815
F1P0L8	Q16850	CYP51A1	NM_000786	77.381
F1NY25	P21399	ACO1	NM_002197	87.514
F1NNE7	Q9UIQ6	LNPEP	NM_175920	67.122
F1NIS1	Q9NYZ3	GTSE1	NM_016426	30.485
F1NBJ2	P63151	PPP2R2A	NM_002717	97.357
E1BVT6	Q96KM6	ZNF512B	NM_020713	21.306
F1N843	P29350	PTPN6	NM_080548	79.605
E1C6A1	Q13423	NNT	NM_182977	88.582
E1BW90	Q96EA4	CCDC99	NM_017785	57.903
E1BVI0	Q9GZX7	AICDA	NM_020661	89.899
E1BUT6	Q75QN2	INTS8	NM_017864	89.447
E1BTY4	Q9Y5X1	SNX9	NM_016224	83.775
P39963	Q8WWL7	CCNB3	NM_033670	14.204
F1NID1	Q6NSI4	CXorf57	NM_018015	40.704
E1C640	Q9UGU5	HMGXB4	NM_001003681	69.421
E1BZR5	Q9BYV9	BACH2	NM_021813	79.097
E1C1R6	O60293	ZFC3H1	NM_144982	72.361
F1NLI8	O95235	KIF20A	NM_005733	68.147
F1NIG4	Q9Y448	C15orf23	NM_033286	27.660
